# Magnesium deficiency score predicts erectile dysfunction risk and mortality: a population-based analysis of NHANES 2001–2004

**DOI:** 10.3389/fnut.2025.1676413

**Published:** 2025-10-31

**Authors:** Xiaobao Chen, Kangqiang Weng, Ruoyun Xie, Junwei Lin, Lingjun Liu, Shaoxing Zhu, Huaiying Zheng

**Affiliations:** Department of Urology, Fujian Medical University Union Hospital, Fuzhou, China

**Keywords:** erectile dysfunction, sexual dysfunction, magnesium deficiency, mortality, NHANES

## Abstract

**Background:**

Magnesium deficiency is associated with numerous cardiovascular and metabolic disorders, which are established risk factors for erectile dysfunction (ED). However, the role of the magnesium depletion score (MDS)—a composite measure of magnesium status—in relation to ED and subsequent mortality remains unclear.

**Methods:**

We analyzed data from 3,917 participants in the National Health and Nutrition Examination Survey (NHANES) 2001–2004. Weighted multivariable logistic and Cox proportional hazards models were used to examine associations between MDS and ED and between MDS and mortality, respectively. Sensitivity analyses included subgroup analyses and propensity score matching.

**Results:**

Of the participants, 1,090 were identified with ED. During follow-up, 654 deaths occurred. After full adjustment, each 1-point increase in MDS was associated with a 37% higher prevalence of ED (OR = 1.37, 95% CI: 1.16–1.62). Among those with ED, higher MDS was also associated with a 30% increased risk of all-cause mortality (HR = 1.30, 95% CI: 1.17–1.45). Sensitivity analyses supported the robustness of these findings.

**Conclusion:**

Higher MDS is significantly associated with both increased prevalence of ED and greater all-cause mortality in men with ED. These results underscore the importance of assessing magnesium status as a potential target for risk stratification and nutritional intervention in the management of ED.

## Introduction

Erectile dysfunction is a prevalent condition among men, particularly those over the age of 40, and it has significant implications for quality of life and overall health (1). ED is characterized by the persistent inability to achieve or maintain an erection sufficient for satisfactory sexual performance. The etiology of ED is multifactorial (2–9), with vascular, neurogenic, hormonal, and psychological factors all playing roles. In recent years, there has been increasing interest in the role of micronutrients, including magnesium, in the pathophysiology of ED (10).

Magnesium is an essential mineral involved in numerous physiological processes, including the metabolism of nitric oxide, a critical mediator of penile erection (11). Hypomagnesemia, or magnesium deficiency, has been implicated in various cardiovascular and metabolic disorders, which are also risk factors for ED. Despite the biological plausibility linking magnesium status to erectile function, the relationship between magnesium deficiency and ED has not been extensively studied.

Previous research has shown that hypomagnesemia is associated with an increased prevalence of ED in certain populations. For instance, a study on elderly men with chronic kidney disease found that those with hypomagnesemia had a significantly higher prevalence of ED compared to those with normal magnesium levels (10). Additionally, dietary intake of magnesium and other trace metals has been inversely associated with the prevalence of ED, suggesting that adequate intake of these nutrients may be protective against the development of ED (12). The concept of a Magnesium Depletion Score (MDS) has been proposed as a composite measure to assess magnesium status in individuals. Conventional methods rely on serum magnesium levels, which may not accurately reflect total body magnesium content, as serum levels constitute only 0.3% of total body magnesium (13). This discrepancy can lead to underdiagnosis of chronic magnesium deficiency, even when serum levels appear normal. The Magnesium Tolerance Test (MTT) is recognized as a reliable method for assessing magnesium status, but its complexity and procedural requirements limit its clinical use (14, 15). In response, Fan et al. (16). Developed the MDS, offering a more accessible and accurate tool for identifying magnesium deficiency. The MDS considers factors such as diuretic and proton pump inhibitor usage, changes in renal function, and alcohol consumption, providing a comprehensive assessment that surpasses the limitations of serum and urinary magnesium measures. Understanding the relationship between MDS and ED could provide insights into potential preventive and therapeutic strategies for ED.

Furthermore, magnesium deficiency has been linked to increased all-cause mortality, highlighting the broader health implications of maintaining adequate magnesium levels (17). Given the potential impact of magnesium on both erectile function and overall mortality, it is important to investigate these relationships comprehensively.

This study aims to explore the association between ED and MDS, utilizing data from the NHANES conducted between 2001 and 2004. Additionally, we will analyze the relationship between MDS and all-cause mortality, providing a holistic view of the health implications of magnesium deficiency. By leveraging a large, nationally representative dataset, this research seeks to contribute to the understanding of the role of magnesium in erectile function and overall health.

## Materials and methods

### Study population

This investigation employed data sourced from the NHANES conducted during the period from 2001 to 2004. The cohort consisted of individuals who had complete datasets pertaining to ED and MDS. Trained interviewers conducted extensive family interviews to collect relevant information, encompassing demographic characteristics, educational attainment, and personal medical histories. Individuals lacking complete demographic data, clinical outcomes, or laboratory results were excluded from the analysis. Ethical approval was secured, along with informed consent from the National Center for Health Statistics Research Ethics Review Board, which was duly communicated to all participants involved. Rigorous procedures were established for data collection and definition.

### Assessment of ED

During the NHANES data collection period, interviews with participants were conducted in private rooms within the Mobile Examination Center (MEC). ED was assessed using the Audio Computer-Assisted Self-Interview (ACASI) method. Participants were asked to describe their ability to achieve and maintain satisfactory erectile function, with response options ranging from “always or almost always able to,” “usually able to,” “sometimes able to,” to “unable to” Participants who reported being “sometimes able to” or “unable to” in maintaining an erection were classified as having erectile dysfunction, while those who reported “always or almost always able to” or “usually able to” were considered not to have erectile dysfunction. This assessment question was adapted from the Massachusetts Male Aging Study (18).

All individuals who fulfilled the eligibility requirements and possessed adequate identifying information were connected with mortality records. This linkage was performed by the research data center at the National Center for Health Statistics, employing the National Death Index. The connection was established using the Linked Mortality Files, which are accessible to the public through the National Center for Health Statistics. The main emphasis of the outcomes was on fatalities attributed to any cause. The follow-up period commenced upon the completion of the NHANES questionnaire and concluded either at the time of death or on December 31, 2019.

### Assessment of MDS

The MDS was calculated following the validated methodology established by Fan et al. (16), which quantifies the cumulative burden of factors known to influence magnesium homeostasis. The score incorporates four key determinants: (1) Diuretic use: current use of any diuretic medication was assigned 1 point, (2) Proton pump inhibitor (PPI) use: current use of any PPI was assigned 1 point, (3) Kidney function: participants with mildly reduced renal function [60 mL/(min·1.73 m^2^) ≤ eGFR <90 mL/(min·1.73 m^2^)] received 1 point; those with more significantly impaired renal function [eGFR < 60 mL/(min·1.73 m^2^)] received 2 points, and (4) Alcohol consumption: participants exceeding recommended consumption thresholds (>1 drink/day for women and >2 drinks/day for men) received 1 point. Current medication use was operationally defined as self-reported use within the preceding 30 days. A standard alcoholic drink was defined according to national guidelines as containing 14 grams of ethanol. The estimated glomerular filtration rate (eGFR) of participants was determined using the Chronic Kidney Disease Epidemiology Collaboration (CKD-EPI) equation (19). The renal function was categorized into three groups: normal renal function [eGFR ≥ 90 mL/(min 1.73 m^2^)], mild renal impairment [60 mL/(min 1.73 m^2^) ≤ eGFR < 90 mL/(min 1.73 m^2^)], and chronic kidney disease [eGFR < 60 mL/(min 1.73 m^2^)]. For analytical purposes, participants were stratified based on their composite MDS as follows: MDS = 0 (no risk factors), MDS = 1 (one risk factor), MDS = 2 (two risk factors), and MDS ≥ 3 (three or more risk factors).

### Covariates

The collection of variables in this study includes demographic characteristics such as age, BMI, marital status, education level, race, and family income. Lifestyle habits like physical activity and smoking status are also considered, along with dietary consumption including dietary magnesium intake and total energy intake. Additionally, comorbid conditions such as coronary heart disease, hypertension, metabolic syndrome, anemia, and hypertension are taken into account, as well as laboratory biochemical indicators.

Diabetes was diagnosed based on a glycosylated hemoglobin (HbA1c) level ≥6.5%, fasting blood glucose level ≥126 mg/dL, use of antidiabetic medications, or self-report. Diagnosis of hypertension was based on systolic/diastolic blood pressure values ≥140/90 mmHg, use of antihypertensive medications, or self-report. Diagnoses of coronary artery disease included heart failure, coronary artery disease, angina, or myocardial infarction. Anemia is determined by hemoglobin levels, with two groups: no anemia (≥130 g/L) and anemia (<130 g/L).

The identification of metabolic syndrome in adult populations is based on the criteria established by the National Cholesterol Education Program’s Adult Treatment Panel III. This framework stipulates that the diagnosis requires the presence of a minimum of three risk factors, which include: (1) waist circumference ≥102 cm for men and ≥88 cm for women; (2) Serum triglycerides ≥150 mg/dL; (3) HDL cholesterol <40 mg/dL for males and <50 mg/dL for females; (4) Systolic blood pressure ≥130 mmHg or diastolic blood pressure ≥85 mmHg; (5) Fasting blood glucose ≥100 mg/dL.

Information regarding dietary magnesium consumption (mg/day) and overall energy intake was gathered through two separate 24-h dietary recall interviews. These interviews documented the participants’ comprehensive energy and nutrient consumption from all food items and beverages. The first phase of data collection took place at the Mobile Examination Center (MEC), while a subsequent interview was carried out via telephone between 3 to 10 days later. To ensure the objectivity of the results, we computed the mean magnesium intake alongside the total energy intake for each participant, utilizing the data obtained from both interview sessions.

### Statistical analysis

In the course of our data analysis, we adhered to the analytical protocols established by the NHANES. Importantly, none of the variables examined in our study exhibited missing data that surpassed the threshold of 10%. To accommodate the intricate sampling design inherent to NHANES, all statistical analyses were conducted utilizing the designated sample weights. We calculated weighted means along with standard errors for continuous variables, and comparisons were performed employing either Student’s t-test or one-way analysis of variance. For categorical data, we presented the results as weighted percentages accompanied by standard errors, and intergroup comparisons were executed using chi-square tests.

A comprehensive weighted multivariable logistic regression analysis was performed to explore the association between MDS and ED. The analysis included adjustments for sociodemographic characteristics, lifestyle behaviors, dietary factors, comorbidities, and laboratory data. To evaluate potential dose–response relationships across MDS categories, we conducted trend tests by incorporating the ordinal MDS variable (0, 1, 2, ≥3) as a continuous term in our regression models. The resulting *p*-value for this term—reported as p for trend—assesses the statistical significance of the linear relationship between increasing MDS categories and the odds of erectile dysfunction. In addition, a multivariable weighted Cox proportional hazards regression analysis was utilized to investigate the relationship between MDS and all-cause mortality. Moreover, weighted Kaplan–Meier curves and log-rank tests were used to analyze cumulative survival differences across different MDS categories.

To ensure the robustness of our study results, we conducted several sensitivity tests. Initially, MDS was categorized as a categorical variable, with MDS = 0 as the reference category, to assess distinct patterns in the relationships. Subsequently, the population was stratified based on factors such as diabetes, cardiovascular diseases, hypertension, anemia, cancer, metabolic syndrome, and magnesium intake for subgroup analysis. Interaction tests were utilized to evaluate heterogeneity among different subgroups. Furthermore, different definitions of ED were employed for analysis, distinguishing participants who were unable or sometimes able to achieve and maintain satisfactory erections for sexual intercourse as having mild or severe erectile dysfunction. Lastly, the MDS was split into two groups: MDS = 0 and MDS > 0. Propensity score matching analysis was then conducted to further investigate the relationship between MDS and ED, as well as the survival outcomes in patients with ED.

All analyses were performed using the statistical software packages R (R version 4.2.0) and Free Statistics software version 1.9.

## Results

### Participant characteristics

**Figure 1:**
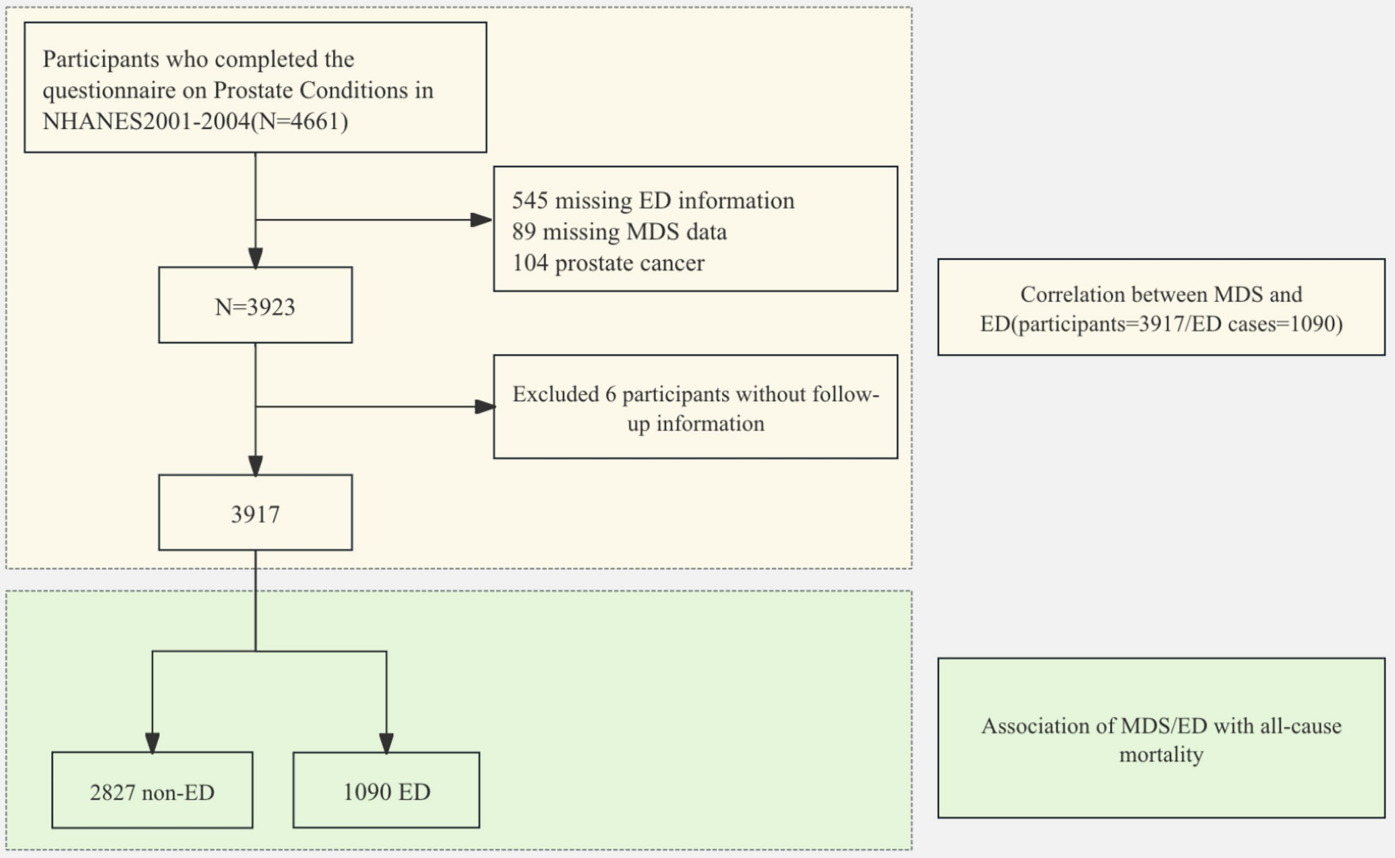
Flowchart of the sample selection from the National Health and Nutrition Examination Survey 2001–2004.

**Figure 2:**
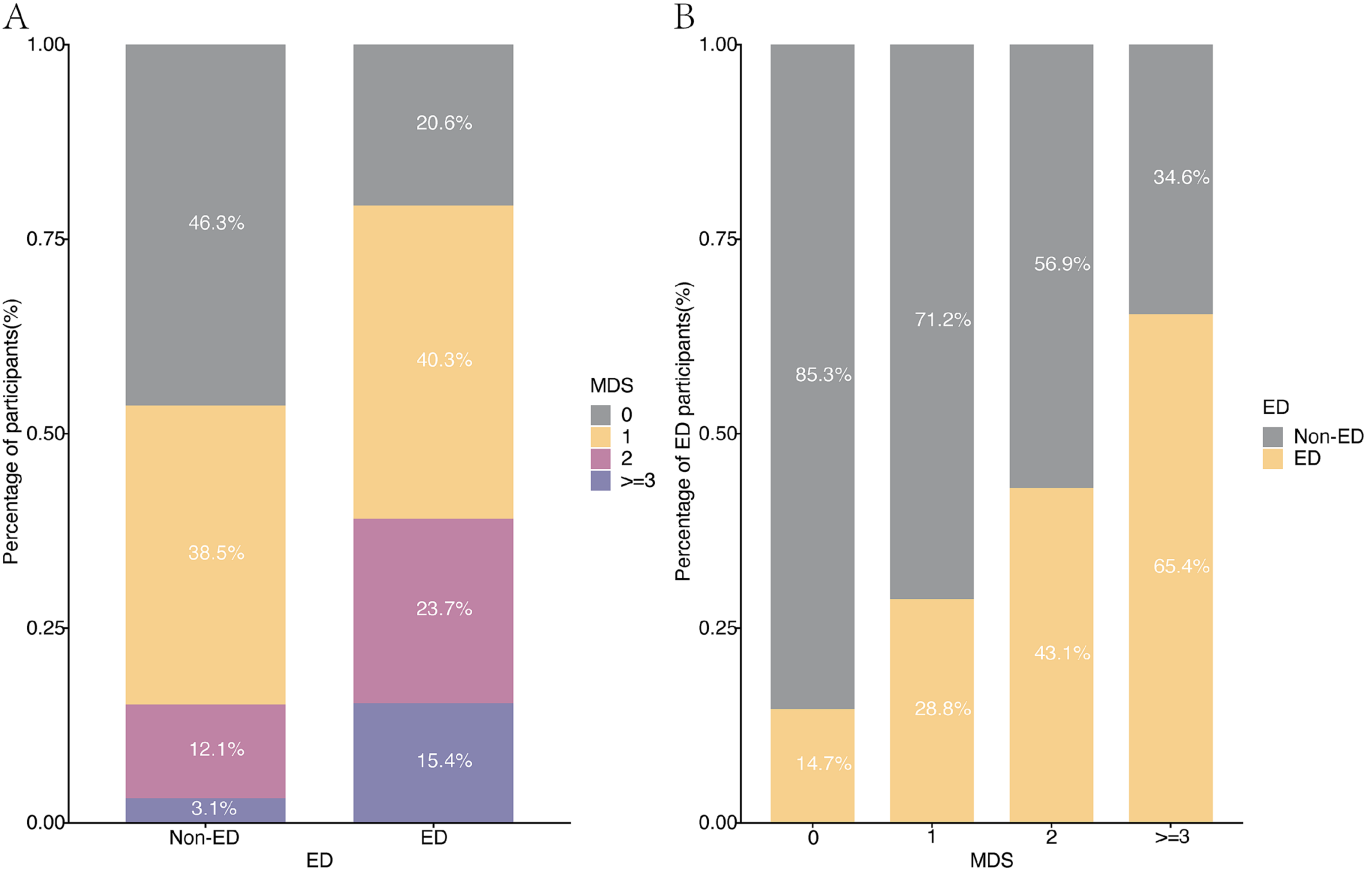
The distribution of MDS and ED in different groups. **(A)** The MDS distribution in non-ED and ED groups. **(B)** The non-ED and ED distribution in different MDS groups.

Participant demographic characteristics are summarized in Table 1, with unweighted data provided in Supplementary Table 1. Following screening, 3,917 cases fulfilled the criteria (Figure 1). The MDS for the ED group was 1.32 ± 0.05, with a magnesium intake of 291.74 ± 7.17 mg. In contrast, the non-ED group exhibited an MDS of 0.72 ± 0.03, with an intake of 336.22 ± 4.32 mg. The proportion of older adults and cohabitants is higher in the ED group compared to the non-ED group. Furthermore, the ED group exhibits higher proportions of elevated BMI, smoking rate, diabetes, cardiovascular disease, hypertension, cancer, anemia, and MetS. Additionally, the MDS in the ED group is significantly higher than that in the non-ED group (Figure 2A). Within each subgroup of MDS, the prevalence of ED increased significantly with higher MDS (Figure 2B).

**Table 1:** Demographic and clinical parameters according to MDS.

Variable	Total	MDS = 0	MDS = 1	MDS = 2	MDS > =3	*p*-value
Age (years)	44.72 ± 0.38	37.27 ± 0.46	46.49 ± 0.47	54.46 ± 0.73	65.41 ± 1.23	<0.0001
Age, n (%)						<0.0001
<40 years	40.45 (0.02)	59.70 (1.93)	33.53 (1.40)	16.64 (2.27)	3.30 (1.64)	
≥40 years	59.55 (0.03)	40.30 (1.93)	66.47 (1.40)	83.36 (2.27)	96.70 (1.64)	
Race, n (%)						<0.0001
Non-Hispanic White	73.72 (0.05)	64.28 (2.35)	78.31 (2.04)	83.51 (2.14)	87.41 (2.20)	
Non-Hispanic Black	9.83 (0.01)	12.02 (1.67)	8.60 (1.01)	7.79 (1.22)	7.39 (1.72)	
Mexican American	7.84 (0.01)	11.83 (1.25)	6.26 (1.15)	2.95 (0.70)	1.32 (0.53)	
Other Race	8.60 (0.01)	11.87 (1.66)	6.83 (1.27)	5.75 (1.54)	3.88 (1.80)	
Marital status, n (%)						<0.001
Solitude	30.73 (0.01)	34.50 (1.85)	30.49 (1.35)	24.38 (2.12)	19.71 (3.02)	
Cohabitation	69.16 (0.04)	65.50 (1.85)	69.51 (1.35)	75.62 (2.12)	80.29 (3.02)	
PIR, n (%)						<0.0001
<1.3	16.01 (0.01)	22.44 (1.52)	14.01 (1.29)	11.20 (1.63)	8.75 (1.74)	
1.3 ~ 3.5	33.86 (0.02)	37.11 (1.98)	34.54 (1.48)	31.60 (2.95)	44.66 (4.06)	
≥3.5	45.14(0.02)	40.45 (2.02)	51.45 (1.88)	57.19 (3.48)	46.58 (4.41)	
BMI, n (%)						<0.001
<25 kg/m^2^	29.20(0.02)	33.94 (1.50)	28.22 (1.24)	24.71 (2.47)	18.27 (2.52)	
25 ~ 30 kg/m^2^	40.52 (0.02)	36.49 (1.75)	43.25 (1.67)	47.35 (2.23)	43.68 (4.62)	
≥30 kg/m^2^	28.89 (0.01)	29.58 (1.63)	28.53 (1.19)	27.95 (2.65)	38.05 (3.43)	
Education level, n (%)						0.13
Less than or high school	43.85 (0.03)	46.21 (1.53)	41.89 (1.71)	41.48 (2.85)	47.97 (4.90)	
Above high school	56.09(0.02)	53.79 (1.53)	58.11 (1.71)	58.52 (2.85)	52.03 (4.90)	
Smoking status, n (%)						<0.0001
Never	42.90 (0.02)	47.54 (2.16)	42.17 (1.72)	35.35 (2.62)	31.69 (3.79)	
Former	28.78 (0.02)	21.34 (1.31)	29.33 (1.22)	40.46 (2.55)	54.05 (4.89)	
Current	28.30 (0.02)	31.12 (1.63)	28.50 (1.29)	24.19 (2.51)	14.25 (3.50)	
Vigorous activity						<0.0001
No	57.64 (0.03)	54.86 (1.76)	56.41 (1.41)	64.05 (2.72)	73.29 (3.41)	
Yes	39.48 (0.02)	44.17 (1.83)	40.69 (1.51)	29.90 (2.02)	16.82 (2.75)	
Unable to do activity	2.88 (0.00)	0.96 (0.24)	2.91 (0.50)	6.05 (1.32)	9.89 (1.93)	
Moderate activity						<0.0001
No	42.28 (0.02)	44.89 (1.67)	40.00 (1.62)	40.51 (2.57)	45.02 (3.05)	
Yes	55.76 (0.03)	54.26 (1.63)	58.06 (1.56)	56.30 (2.83)	47.85 (2.94)	
Unable to do activity	1.90 (0.00)	0.85 (0.23)	1.93 (0.36)	3.18 (1.02)	7.13 (1.85)	
DM, n (%)						<0.0001
No	89.62 (0.04)	91.37 (0.88)	91.24 (0.85)	85.54 (1.82)	72.36 (3.29)	
Yes	10.38 (0.01)	8.63 (0.88)	8.76 (0.85)	14.46 (1.82)	27.64 (3.29)	
CVD, n (%)						<0.0001
No	90.96 (0.04)	96.57 (0.67)	92.19 (0.69)	81.82 (1.84)	58.89 (4.05)	
Yes	9.02 (0.01)	3.43 (0.67)	7.81 (0.69)	18.18 (1.84)	41.11 (4.05)	
Hypertension, n (%)						<0.0001
No	65.53 (0.03)	77.54 (1.32)	65.51 (2.06)	46.58 (2.60)	19.45 (3.33)	
Yes	34.33 (0.02)	22.46 (1.32)	34.49 (2.06)	53.42 (2.60)	80.55 (3.33)	
Anemia, n (%)						<0.0001
No	95.00 (0.04)	99.02 (0.24)	97.46 (0.40)	95.99 (0.78)	88.26 (1.72)	
Yes	2.49(0.00)	0.98(0.24)	2.54(0.40)	4.01(0.78)	11.74(1.72)	
Cancer, n (%)						<0.0001
No	93.56 (0.04)	97.86 (0.39)	93.05 (0.59)	87.52 (1.48)	79.83 (2.20)	
Yes	6.37 (0.01)	2.14 (0.39)	6.95 (0.59)	12.48 (1.48)	20.17 (2.20)	
MetS, n (%)						<0.0001
No	74.66 (0.03)	79.31 (1.54)	76.31 (1.23)	66.49 (2.63)	43.99 (4.28)	
Yes	25.34 (0.02)	20.69 (1.54)	23.69 (1.23)	33.51 (2.63)	56.01 (4.28)	
ED, n (%)						<0.0001
No	81.36 (0.03)	89.83 (0.85)	81.31 (1.37)	70.27 (2.00)	40.48 (4.35)	
Yes	18.64 (0.01)	10.17 (0.85)	18.69 (1.37)	29.73 (2.00)	59.52 (4.35)	
Magnesium intake (mg)	327.93 ± 4.24	324.23 ± 5.75	339.38 ± 6.93	321.59 ± 6.62	280.23 ± 10.60	<0.001
Albumin (g/L)	43.83 ± 0.08	44.22 ± 0.11	43.82 ± 0.09	43.21 ± 0.20	42.45 ± 0.25	<0.0001
Total energy (kcal)	2666.63 ± 25.49	2695.43 ± 40.01	2735.59 ± 46.58	2571.18 ± 47.38	2098.63 ± 65.44	<0.0001

Values are mean ± SD (continuous variables) or n% (categorical variables) are weighted. BMI, Body mass index; PIR, Poverty to income ratio; eGFR, estimated Glomerular Filtration Rate; PPI, Proton Pump Inhibitors; DM, Diabetes mellitus; CVD, Cardiovascular disease; MetS, Metabolic syndrome; MDS, Magnesium Deficiency Scores.

### The association between MDS and ED

Table 2 displays the outcomes of the weighted logistic regression analysis examining the relationship between MDS and ED. The findings reveal a positive association between MDS and ED across various adjusted models. In the unadjusted model, the ORs were 2.11 (95% CI: 1.88–2.35, *p* < 0.001). Following adjustment for all covariates, the ORs decreased to 1.41 (95% CI: 1.20–1.67, *p* < 0.001).

**Table 2:** Association between MDS and ED.

Characteristic	crude model OR (95%CI)	Model 1 OR (95%CI)	Model 2 OR (95%CI)	Model 3 OR (95%CI)
MDS continue	2.11 (1.88, 2.36)	1.76 (1.56, 1.97)	1.62 (1.44, 1.84)	1.37 (1.16, 1.62)
MDS category				
0	Ref	Ref	Ref	Ref
1	2.03 (1.58, 2.61)	1.67 (1.27, 2.19)	1.58 (1.19, 2.10)	1.50 (0.84, 2.66)
2	3.75 (2.80, 5.04)	2.68 (1.88, 3.81)	2.28 (1.57, 3.31)	1.78 (0.87, 3.61)
≥3	12.99 (8.47, 19.91)	6.95 (4.37, 11.03)	5.50 (3.41, 8.90)	3.01 (1.10, 8.21)
*p* for trend	<0.0001	<0.0001	<0.0001	0.01

Unadjusted model: no covariates were adjusted. Model 1, age, race, marital status, education, FIR and BMI were adjusted. Model 2, Model 1 + alcohol intake, smoking status, vigorous and moderate activity were adjusted. Model 3, Model 2+, DM, CVD, hypertension, CKD, cinema, cancer, MetS, albumin, magnesium intake and energy were adjusted.

To further analyze the data, we stratified the MDS into comparative groups, establishing the MDS = 0 category as the reference point. In the unadjusted model, the OR demonstrated a significant increase, ranging from 2.03 (95% CI: 1.58–2.61) to a notably higher value of 12.99 (95% CI: 8.47–19.91). Upon adjustment, the increment in OR was attenuated; however, it still exhibited a substantial rise from 1.50 (95% CI: 0.84–2.66) to 3.01 (95% CI: 1.1–8.21). To quantify the potential impact of unmeasured confounding, we calculated E-values for the fully adjusted associations. The E-value for the continuous MDS (OR = 1.37) was 1.61, indicating that an unmeasured confounder would need to be associated with both MDS and ED by risk ratios of at least 1.61-fold to explain away the observed association. For categorical MDS, the E-values were 1.74 (MDS = 1), 2.00 (MDS = 2), and 2.86 (MDS ≥ 3), suggesting that relatively strong confounding would be necessary to nullify these associations. This pattern of correlation remained consistent across various adjusted models, confirming a linear trend that achieved statistical significance (*p* for trend < 0.05).

### Subgroup analysis

Table 3 delineates the intricate results of subgroup analyses and interaction tests, which explore the influence of stratification on the correlation between MDS and ED. The data consistently reveal a positive association between MDS and ED across all examined subgroups, with this relationship persisting even after meticulous adjustments for potential confounding covariates. In the realm of interaction effects, a noteworthy interaction was identified solely in the context of cancer history, with statistical significance indicated by a *p*-value less than 0.05. Conversely, no such interactions were observed for the remaining variables. The subgroup analysis, particularly among participants without a history of cancer, demonstrates that those with an MDS of ≥3 are at a significantly elevated risk of ED, with a 2.68-fold increase compared to those with an MDS of 0 (95% CI: 1.83–7.41). Furthermore, a compelling linear relationship between the MDS and the risk of ED is evident, underscored by a highly significant *p*-value for the trend test (*p* < 0.001). Regarding trend tests, the linear association was found to be non-significant for anemia, cancer, and high levels of magnesium intake, as indicated by a *p*-value exceeding 0.05. However, for all other variables, a pronounced linear relationship is discernible.

**Table 3:** Subgroup analysis of the association between MDS and ED.

Subgroup	MDS = 0	MDS = 1	MDS = 2	MDS ≥3	*p* for trend	*P* for interaction
CVD						0.4
No	Ref	1.55 (1.10, 2.18)	1.91 (1.28, 2.84)	2.51 (1.24, 5.08)	0.001	
Yes	Ref	1.63 (0.54, 4.91)	2.07 (0.45, 9.43)	7.91 (2.09, 29.92)	0.01	
DM						0.8
No	Ref	1.52 (1.13, 2.05)	1.78 (1.27, 2.50)	3.44 (2.00, 5.91)	<0.001	
Yes	Ref	1.74 (0.78, 3.89)	2.85 (1.04, 7.82)	3.34 (0.78, 14.38)	0.04	
Hypertension						0.2
No	Ref	1.27 (0.86, 1.88)	1.71 (1.00, 2.91)	3.34 (0.95, 11.69)	0.01	
Yes	Ref	2.16 (1.42, 3.28)	2.39 (1.37, 4.16)	4.08 (2.32, 7.16)	<0.001	
Anemia						0.41
No	Ref	1.56 (1.15, 2.12)	2.00 (1.39, 2.90)	3.59 (1.99, 6.50)	<0.001	
Yes	Ref	0.90 (0.11, 7.67)	1.13 (0.08, 16.73)	1.28 (0.13, 13.17)	0.68	
Cancer						0.01
No	Ref	1.39 (1.00, 1.93)	1.80 (1.21, 2.67)	3.68 (1.83, 7.41)	<0.001	
Yes	Ref	9.89 (1.58, 62.12)	7.51 (1.23, 45.71)	7.34 (1.01, 53.02)	0.19	
MetS						0.53
No	Ref	1.58 (0.99, 2.51)	1.73 (0.92, 3.27)	2.61 (1.13, 6.03)	0.04	
Yes	Ref	1.55 (1.07, 2.23)	2.07 (1.35, 3.18)	4.56 (2.08, 10.03)	<0.001	
Dietary magnesium intake						0.93
Q1	Ref	1.58 (0.92, 2.73)	2.63 (1.57, 4.40)	3.45 (1.60, 7.46)	<0.001	
Q2	Ref	1.49 (0.92, 2.43)	1.74 (0.99, 3.03)	3.43 (1.27, 9.26)	0.01	
Q3	Ref	1.45 (0.69, 3.05)	1.40 (0.57, 3.45)	3.26 (1.01, 10.56)	0.08	

Unadjusted model: no covariates were adjusted. Model 1, age, race, marital status, education, FIR and BMI were adjusted. Model 2, Model 1 + alcohol intake, smoking status, vigorous and moderate activity were adjusted. Model 3, Model 2+, DM, CVD, hypertension, CKD, Anemia, cancer, MetS, albumin, magnesium intake and energy were adjusted. Analysis was stratified by diabetes, CVD, CKD, hypertension, cancer, anemia, MetS, and dietary magnesium intake, not adjusted for the stratification variable itself.Note: Subgroup analyses were performed under model 3, adjusting for age, race, marital status, education, FIR, BMI, alcohol intake, smoking status, vigorous and moderate physical activity, DM, CVD, hypertension, CKD, anemia, cancer, MetS, serum albumin, dietary magnesium intake, and total energy intake; the stratification variable itself was not adjusted for.

### Sensitivity analysis

To further minimize the impact of confounding variables, we utilized PSM analysis to categorize MDS into MDS = 0 and MDS > 0 groups. The findings (Supplementary Table 2) consistently reveal a significant association between MDS and ED across different propensity score techniques. Specifically, using the IPTW method, the likelihood of developing ED was found to be 67% higher in the MDS > 0 group compared to the MDS = 0 group (OR 1.67, 95% CI 1.41–1.97).

### The association of mortality with different MDS and ED status

**Figure 3:**
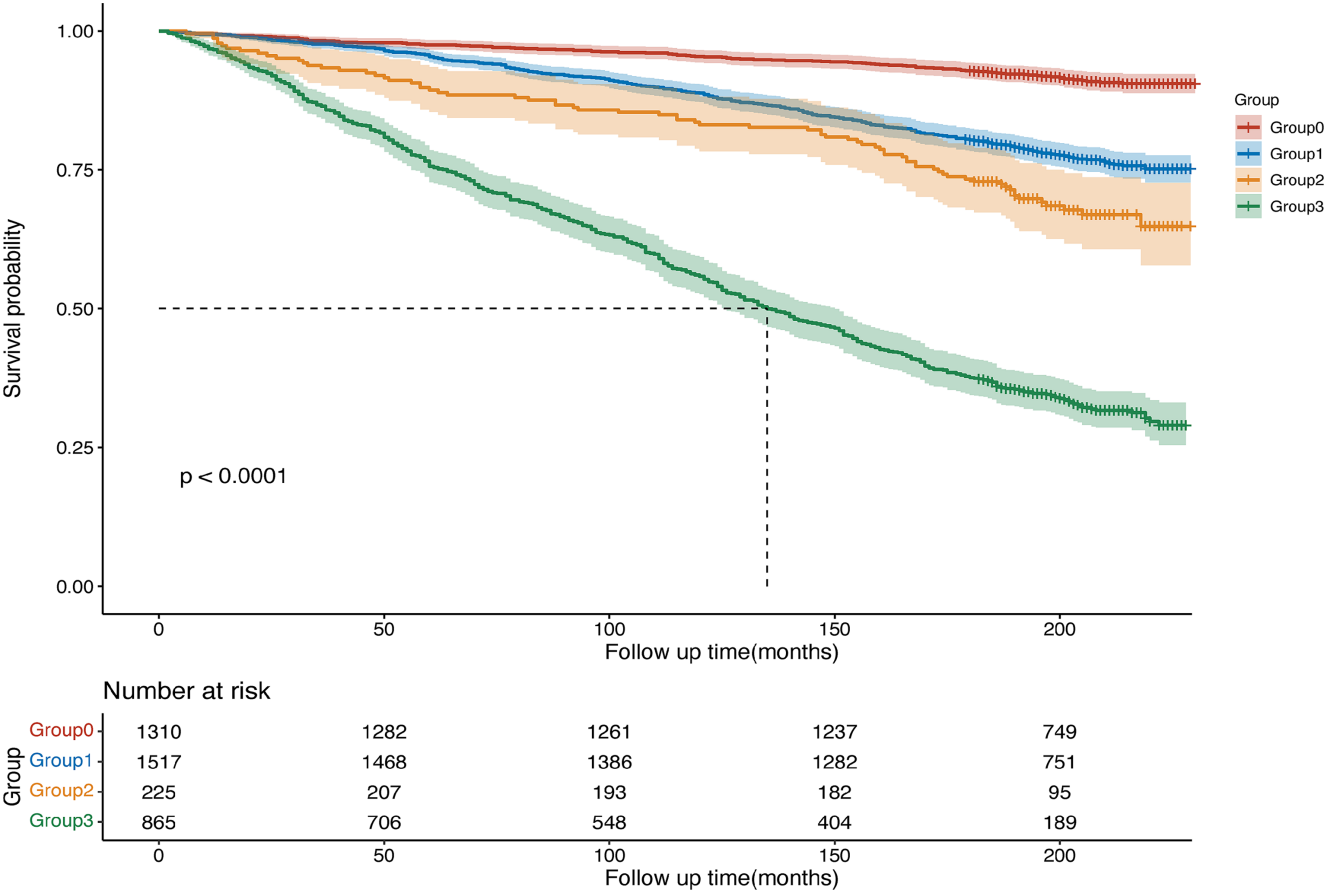
Kaplan–Meier curves were used to present the status of MDS and ED with all-cause mortality.

**Figure 4:**
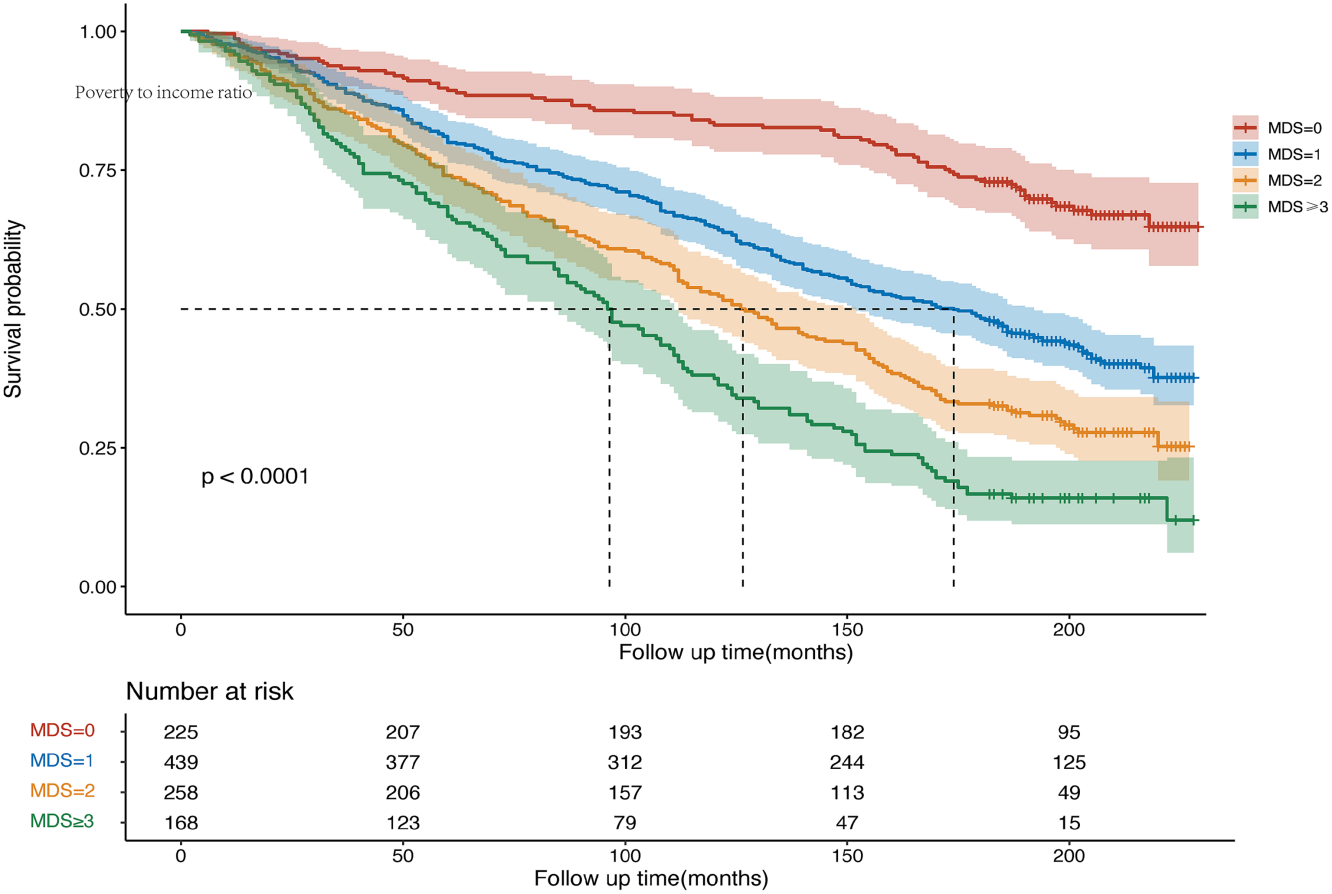
Kaplan-Maier cures were used to present the relationship of the MDS with all-cause mortality among participants with ED.

Over a median follow-up period of 206 months, our study meticulously identified 1,116 cases of all-cause mortality within the entire cohort, with a notable 654 cases occurring in the ED group. The baseline characteristics of the ED participants, categorized by overall survival (OS), are succinctly detailed in Supplementary Table 3. A rigorous multifactorial Cox regression analysis revealed significant prognostic disparities associated with various combinations of MDS and ED status. Specifically, the prognosis for the MDS > 0 and ED group was markedly poorer compared to the MDS = 0 and non-ED group, with a HR of 2.18 (95% CI, 1.61–2.96) (Table 4). The Kaplan–Meier curves, delineated in Figure 3, vividly portray the survival rates for all-cause mortality across these distinct MDS and ED groupings. Further assessment of the influence of MDS on prognosis within the ED group, outlined in Table 5, underscored a direct correlation that higher MDS were linked to a more adverse prognosis. After accounting for various confounding factors, individuals with MDS of 1, 2, and ≥3 were observed to have a 98, 161, and 179% increased risk of all-cause mortality, respectively, compared to those with an MDS of 0 (HR 1.98, 95% CI 1.22–3.21; HR 2.61, 95% CI 1.58–4.3; HR 2.79, 95% CI 1.73–4.49). The Kaplan–Meier curves, elegantly depicted in Figure 4, illustrate the stark survival differences based on MDS. Notably, participants with MDS of ≥3 exhibited the lowest survival rate, while those with scores of 1 or 2 demonstrated an intermediate rate. In contrast, participants with an MDS of 0 enjoyed the highest survival rate, with the survival disparities being statistically significant as indicated by the log-rank test (*p* < 0.001).

**Table 4:** The Association of mortality with different statuses of MDS and ED.

Characteristic	Crude model HR (95%CI)	*P*-value	Adjusted model HR (95%CI)	*P*-value
Groups				
Group 0	Ref		Ref	
Group 1	2.10 (1.66, 2.66)	<0.0001	1.19 (0.88, 1.61)	0.27
Group 2	3.38 (2.24, 5.10)	<0.0001	1.00 (0.60, 1.68)	0.99
Group 3	11.13 (8.85, 13.99)	<0.0001	2.18 (1.61, 2.96)	<0.0001
*p* for trend	<0.0001		<0.0001	

Crude model: no covariates adjusted. Adjusted model: adjusted for age, race, marital status, education, FIR and BMI, alcohol intake, smoking status, vigorous and moderate activity, DM, CVD, hypertension, Anemia, cancer, MetS, albumin, magnesium intake and energy. Group 0: MDS = 0 and non-ED; Group 1: MDS>0 and non-ED; Group 2: MDS = 0 and ED; Group 3: MDS>0 and ED.

**Table 5:** The association between MDS and mortality in ED.

Characteristic	Crude model HR (95%CI)	*P*-value	Adjusted model HR (95%CI)	*P-*value
All-cause mortality				
MDS continue	1.60 (1.42, 1.79)	<0.0001	1.30 (1.17, 1.45)	<0.0001
MDS category				
0	Ref		Ref	Ref
1	2.46 (1.69, 3.59)	<0.0001	1.98 (1.22, 3.21)	0.01
2	3.69 (2.47, 5.49)	<0.0001	2.61 (1.58, 4.30)	<0.001
≥3	5.51 (3.51, 8.64)	<0.0001	2.79 (1.73, 4.49)	<0.0001
*p* for trend	<0.0001		<0.0001	

Crude model: no covariates adjusted. Adjusted model: adjusted for age, race, marital status, education, FIR and BMI, alcohol intake, smoking status, vigorous and moderate activity, DM, CVD, hypertension, Anemia, cancer, MetS, albumin, magnesium intake and energy.

To address potential confounding factors, PSM was conducted as a sensitivity analysis (Supplementary Table 4). The consistent findings across various PSM methods demonstrated that individuals with MDS > 0 had a 0.67–1.4 times higher risk of all-cause mortality compared to those with MDS of 0.

## Discussion

In this nationwide population-based study, we demonstrate for the first time a significant dose-dependent association between magnesium deficiency score (MDS) and erectile dysfunction (ED), independent of established risk factors. Furthermore, we reveal that concurrent magnesium deficiency substantially amplifies mortality risk among individuals with ED, suggesting important clinical and public health implications. These results highlight the necessity of considering magnesium status in the therapeutic approach to ED and emphasize the potential public health significance of addressing magnesium deficiency.

Our investigation provides novel insights into the complex relationship between magnesium homeostasis and erectile function, addressing a significant knowledge gap in current literature. Previous studies have primarily focused on the role of individual micronutrients in ED or the broader implications of magnesium deficiency on cardiovascular and metabolic health. However, the comprehensive assessment of MDS, which includes factors like diuretic use, proton pump inhibitor use, excessive alcohol consumption, and renal function decline, offers a more holistic view of how magnesium status affects ED. The weighted multivariable logistic regression analysis demonstrating a positive correlation between MDS and ED, even after adjusting for confounding variables, underscores the potential pathophysiological link between magnesium deficiency and erectile function. To our knowledge, this study is the first to systematically quantify both ED risk and mortality outcomes using a composite magnesium deficiency score, providing new insights for risk stratification and targeted intervention strategies.

The clinical implications of our findings are substantial and potentially transformative for ED management. The observed positive correlation between MDS and ED highlights the potential role of magnesium in maintaining vascular health and erectile function. Given that magnesium is involved in numerous physiological processes (20, 21), including vasodilation and endothelial function, its deficiency could exacerbate vascular conditions that contribute to ED. MDS provides a practical clinical tool for identifying patients at risk of magnesium deficiency. Based on our findings, we recommend that patients with MDS ≥ 2 should undergo comprehensive magnesium status evaluation, particularly those with comorbid diabetes, hypertension, or cardiovascular diseases. Our results suggest that addressing magnesium deficiency through dietary modifications or targeted supplementation could represent a novel, modifiable therapeutic target for both ED prevention and mortality reduction. This is particularly relevant for older adults and individuals with chronic health conditions who are at higher risk of both magnesium deficiency and ED. Additionally, the strong association between higher MDS and increased all-cause mortality underscores the importance of comprehensive nutritional assessments in clinical practice. By identifying and correcting magnesium deficiency, healthcare providers may not only improve erectile function but also enhance long-term survival outcomes. Future randomized controlled trials are warranted to determine whether magnesium supplementation can effectively improve erectile function and reduce mortality risk in deficient individuals.

Our subgroup analyses revealed a particularly pronounced association between MDS and ED among cancer survivors, with substantially elevated odds ratios compared to the general population. This pronounced interaction may be explained by several synergistic pathways. Magnesium deficiency is prevalent in cancer patients (22–27) due to multiple mechanisms, including decreased nutritional intake, malabsorption, and increased renal excretion induced by chemotherapeutic agents. The profound magnesium depletion characteristic of cancer populations may exacerbate underlying vascular dysfunction through impaired nitric oxide bioavailability and enhanced oxidative stress (28). Additionally, cancer-related inflammation and endothelial damage likely compound the detrimental effects of magnesium deficiency on erectile function. Furthermore, certain chemotherapeutic agents and analgesics (21) may directly contribute to both magnesium wasting and neurological or vascular erectile dysfunction. Given these synergistic pathways, our findings suggest that magnesium status assessment and correction should be integrated into comprehensive oncological care protocols, not only for general health maintenance but also for preserving sexual function and quality of life in cancer survivors. However, these interpretations should be approached with caution given the relatively small sample size in the cancer subgroup, and further research specifically targeting this population is warranted.

Our mortality analysis employed two complementary analytical approaches that consistently demonstrate the clinical significance of magnesium deficiency. The first approach (Table 4) categorized participants into four groups based on combined MDS and ED status, revealing that individuals with both elevated MDS and ED (Group 3) exhibited significantly higher mortality risk (HR = 2.20, 95% CI: 1.71–2.83) compared to those without either condition (Group 0). This finding suggests a potential synergistic effect between magnesium deficiency and ED on mortality risk. The second approach (Table 5) specifically examined the ED subpopulation, demonstrating a dose–response relationship between MDS severity and mortality, with MDS ≥ 3 associated with the highest risk (HR = 2.79, 95% CI: 1.73–4.49). Both analytical approaches consistently demonstrate that while ED independently increases mortality risk, this association is substantially magnified with concurrent magnesium deficiency, and the risk appears proportional to the severity of magnesium depletion. These findings emphasize the clinical relevance of assessing magnesium status in patients with ED as a potential strategy for improving long-term outcomes.

Several interconnected biological mechanisms may elucidate the observed relationship between magnesium deficiency and erectile dysfunction. Magnesium serves as a critical cofactor for endothelial nitric oxide synthase (eNOS), and its deficiency significantly compromises nitric oxide (NO) production while simultaneously enhancing oxidative stress (21, 29, 30), thereby diminishing the essential vasodilatory capacity required for penile erection. Furthermore, magnesium functions as an endogenous calcium channel antagonist in vascular smooth muscle; consequently, magnesium depletion results in elevated intracellular calcium concentrations, promoting pathological vasoconstriction and impeding the necessary relaxation of corpus cavernosum smooth muscle (21). Magnesium deficiency also induces a pro-inflammatory state characterized by increased levels of inflammatory cytokines and oxidative stress markers, contributing to endothelial dysfunction and vascular impairment specifically within penile microvasculature (31, 32). Compelling evidence indicates magnesium’s regulatory role in testosterone biosynthesis and hormonal homeostasis, both fundamental to normal erectile physiology (33, 34). Additionally, magnesium deficiency is intricately linked with insulin resistance and various components of metabolic syndrome, established independent risk factors for erectile dysfunction (12, 35). Lastly, magnesium modulates autonomic nervous system function, with its deficiency potentially disrupting the parasympathetic predominance essential for initiating and maintaining erection (21). These physiological mechanisms likely operate synergistically in magnesium-deficient states to compromise erectile function, providing robust biological plausibility for the epidemiological associations observed in our study.

This study presents several methodological strengths that enhance the validity and generalizability of our findings. Firstly, it benefits from a large and representative sample of the US population, encompassing two cycles of cross-sectional data. This ample sample size ensures sufficient statistical power for rigorous analysis. Secondly, the application of sampling weights enhances the generalizability and representativeness of the survey findings at a national level. Thirdly, the researchers meticulously accounted for various potential confounding factors, drawing on prior research and clinical expertise to bolster the reliability and validity of the results. Fourthly, the study opted for MDS over serum magnesium, as the former offers a more accurate reflection of magnesium’s physiological status. Lastly, sensitivity analyses were performed to confirm the robustness and reliability of the findings.

Nevertheless, several important limitations warrant consideration in interpreting our results. The primary limitation is the cross-sectional design for the core MDS-ED association, which precludes establishing causal relationships. While our mortality analysis employed a longitudinal approach, the fundamental association between MDS and ED remains correlational. Our findings should therefore be interpreted as indicating associations rather than implying that magnesium deficiency causes ED. Secondly, despite attempts to control for potential confounders, the impact of unmeasured or residual confounders cannot be entirely eradicated. Thirdly, while we rely on MDS as our primary magnesium status indicator based on its theoretical superiority over serum magnesium levels, we cannot empirically validate this assertion within our dataset due to the unavailability of serum magnesium data in the NHANES 2001–2004 cycles. This limitation prevents direct comparison of MDS performance against serum magnesium measurements in our specific population. Additionally, while MDS demonstrates superior predictive ability compared to serum magnesium for magnesium deficiency, both methods have limitations in accurately detecting subclinical magnesium deficiency. This limitation may have resulted in underestimating the true association between magnesium deficiency and ED in our study. Fourthly, participants with MDS ≥ 3 showed substantially increased risk for both ED (adjusted OR = 3.01) and mortality (HR = 2.79), but the corresponding confidence intervals are relatively wide (ED: 1.10–8.21; Mortality: 1.73–4.49), reflecting considerable statistical uncertainty likely due to the small number of participants in this high-risk category. Therefore, while severe magnesium depletion appears associated with significantly increased risk, the precise magnitude should be interpreted cautiously. Lastly, caution should be exercised when extrapolating the study results to other populations, as they may be specific to the US population.

Future research should prioritize two key directions to advance our understanding. First, prospective longitudinal studies measuring magnesium status before ED development are essential to establish temporal relationships and explore potential causal mechanisms. Additionally, randomized controlled trials (RCTs) investigating the effects of magnesium supplementation on ED and overall mortality are warranted. Second, studies with access to both MDS components and serum magnesium data would be valuable to validate the comparative utility of these assessment approaches in relation to ED outcomes. Such studies should also explore the underlying biological mechanisms linking magnesium deficiency to ED and mortality. Moreover, the development and validation of more precise and comprehensive tools for assessing magnesium status in clinical settings could enhance the accuracy of future research. By addressing these gaps, future studies can provide more definitive evidence on the role of magnesium in ED and broader health outcomes, potentially informing clinical guidelines and public health strategies for the prevention and management of magnesium deficiency and its associated risks.

## Conclusion

In conclusion, our findings reveal a significant dose-dependent association between magnesium deficiency score and both erectile dysfunction and subsequent mortality. These robust relationships persist after comprehensive confounder adjustment, indicating that magnesium status represents a potentially modifiable risk factor in erectile physiology and overall health outcomes. While prospective studies are needed to establish causality, our results provide compelling evidence that magnesium deficiency assessment and correction could represent a novel therapeutic approach in ED management and mortality risk reduction. Given the high prevalence of both magnesium deficiency and erectile dysfunction worldwide, these findings have substantial implications for clinical practice and public health strategies.

## Data Availability

The original contributions presented in the study are included in the article/Supplementary material, further inquiries can be directed to the corresponding authors.
