# Supplementary material for: Magnesium deficiency score predicts erectile dysfunction risk and mortality: a population-based analysis of NHANES 2001–2004
Source: Front Nutr. 2025 Oct 31;12:1676413. doi: 10.3389/fnut.2025.1676413 (PMC12616636; doi:10.3389/fnut.2025.1676413)
Supplement: Supplementary file 1 [file Table_1.docx]

**Supplement Table 1 Demographic and clinical parameters according to MDS (unweighted).**

| Variable | Total | MDS=0 | MDS=1 | MDS=2 | MDS>=3 | P value |
| --- | --- | --- | --- | --- | --- | --- |
| Age(years) | 44.72±0.38 | 37.27±0.46 | 46.49±0.47 | 54.46±0.73 | 65.41±1.23 | < 0.0001 |
| Age, n (%) |  |  |  |  |  | < 0.0001 |
| <40 years | 1349(40.45) | 860(59.70) | 418(33.53) | 66(16.64) | 5( 3.30) |  |
| >=40 years | 2574(59.55) | 675(40.30) | 1113(66.47) | 534(83.36) | 252(96.70) |  |
| Race, n (%) |  |  |  |  |  | < 0.0001 |
| Non-Hispanic White | 2105(73.72) | 627(64.28) | 885(78.31) | 400(83.51) | 193(87.41) |  |
| Non-Hispanic Black | 750( 9.83) | 350(12.02) | 264( 8.60) | 100( 7.79) | 36( 7.39) |  |
| Mexican Ameirican | 809( 7.84) | 422(11.83) | 294( 6.26) | 73( 2.95) | 20( 1.32) |  |
| Other Race | 259( 8.60) | 136(11.87) | 88( 6.83) | 27( 5.75) | 8( 3.88) |  |
| Marital status, n (%) |  |  |  |  |  | < 0.001 |
| Solitude | 1257(30.73) | 564(34.50) | 470(30.49) | 155(24.38) | 68(19.71) |  |
| Cohabitation | 2664(69.16) | 970(65.50) | 1060(69.51) | 445(75.62) | 189(80.29) |  |
| PIR, n (%) |  |  |  |  |  | < 0.0001 |
| ＜1.3 | 901(16.01) | 431(22.44) | 320(14.01) | 105(11.20) | 45( 8.75) |  |
| 1.3~3.5 | 1451(33.86) | 575(37.11) | 559(34.54) | 207(31.60) | 110(44.66) |  |
| ≥3.5 | 1357(45.14) | 456(40.45) | 562(51.45) | 250(57.19) | 89(46.58) |  |
| BMI, n (%) |  |  |  |  |  | < 0.001 |
| ＜25kg/m2 | 1142(29.20) | 511(33.94) | 438(28.22) | 141(24.71) | 52(18.27) |  |
| 25~30kg/m2 | 1602(40.52) | 579(36.49) | 650(43.25) | 266(47.35) | 107(43.68) |  |
| ≥30kg/m2 | 1086(28.89) | 412(29.58) | 413(28.53) | 177(27.95) | 84(38.05) |  |
| Education level, n (%) |  |  |  |  |  | 0.13 |
| Less than or high school | 2073(43.85) | 843(46.21) | 784(41.89) | 302(41.48) | 144(47.97) |  |
| Above high school | 1848(56.09) | 691(53.79) | 746(58.11) | 298(58.52) | 113(52.03) |  |
| Smoking status, n (%) |  |  |  |  |  | < 0.0001 |
| Never | 1585(42.90) | 720(47.54) | 591(42.17) | 193(35.35) | 81(31.69) |  |
| Former | 1256(28.78) | 329(21.34) | 517(29.33) | 270(40.46) | 140(54.05) |  |
| Current | 1079(28.30) | 483(31.12) | 423(28.50) | 137(24.19) | 36(14.25) |  |
| Vigorous activity |  |  |  |  |  | < 0.0001 |
| No | 2444(57.64) | 881(54.86) | 954(56.41) | 416(64.05) | 193(73.29) |  |
| Yes | 1328(39.48) | 634(44.17) | 518(40.69) | 147(29.90) | 29(16.82) |  |
| Unable to do activity | 151( 2.88) | 20(0.96) | 59(2.91) | 37(6.05) | 35(9.89) |  |
| Moderate activity |  |  |  |  |  | < 0.0001 |
| No | 1915(42.28) | 793(44.89) | 718(40.00) | 277(40.51) | 127(45.02) |  |
| Yes | 1915(55.76) | 727(54.26) | 780(58.06) | 302(56.30) | 106(47.85) |  |
| Unable to do activity | 92( 1.90) | 14(0.85) | 33(1.93) | 21(3.18) | 24(7.13) |  |
| DM, n (%) |  |  |  |  |  | < 0.0001 |
| No | 3367(89.62) | 1375(91.37) | 1327(91.24) | 484(85.54) | 181(72.36) |  |
| Yes | 556(10.38) | 160( 8.63) | 204( 8.76) | 116(14.46) | 76(27.64) |  |
| CVD, n (%) |  |  |  |  |  | < 0.0001 |
| No | 3403(90.96) | 1471(96.57) | 1349(92.19) | 455(81.82) | 128(58.89) |  |
| Yes | 519( 9.02) | 64( 3.43) | 181( 7.81) | 145(18.18) | 129(41.11) |  |
| Hypertension, n (%) |  |  |  |  |  | < 0.0001 |
| No | 2351(65.53) | 1173(77.54) | 908(65.51) | 230(46.58) | 40(19.45) |  |
| Yes | 1568(34.33) | 360(22.46) | 621(34.49) | 370(53.42) | 217(80.55) |  |
| Anemia, n (%) |  |  |  |  |  | < 0.0001 |
| No | 3623(95.00) | 1424(99.02) | 1443(97.46) | 548(95.99) | 208(88.26) |  |
| Yes | 180( 2.49) | 25( 0.98) | 58( 2.54) | 49( 4.01) | 48(11.74) |  |
| Cancer, n (%) |  |  |  |  |  | < 0.0001 |
| No | 3638(93.56) | 1496(97.86) | 1419(93.05) | 519(87.52) | 204(79.83) |  |
| Yes | 282( 6.37) | 38( 2.14) | 111( 6.95) | 81(12.48) | 52(20.17) |  |
| MetS, n (%) |  |  |  |  |  | < 0.0001 |
| No | 3097(80.56) | 1323(85.66) | 1222(81.58) | 412(71.87) | 140(53.11) |  |
| Yes | 826(19.44) | 212(14.34) | 309(18.42) | 188(28.13) | 117(46.89) |  |
| ED, n (%) |  |  |  |  |  | < 0.0001 |
| No | 2832(81.36) | 1310(89.83) | 1091(81.31) | 342(70.27) | 89(40.48) |  |
| Yes | 1091(18.64) | 225(10.17) | 440(18.69) | 258(29.73) | 168(59.52) |  |
| Magnesium intake(mg) | 327.93±4.24 | 324.23± 5.75 | 339.38± 6.93 | 321.59± 6.62 | 280.23±10.60 | < 0.001 |
| Albumin (g/L) | 43.83±0.08 | 44.22±0.11 | 43.82±0.09 | 43.21±0.20 | 42.45±0.25 | < 0.0001 |
| Total energy (kcal) | 2666.63±25.49 | 2695.43±40.01 | 2735.59±46.58 | 2571.18±47.38 | 2098.63±65.44 | < 0.0001 |

Values are mean +/- SD (continuous variables) or n% (categorical variables) are weighted.

Abbreviation: BMI=Body mass index, PIR=Poverty to income ratio, eGFR=estimated Glomerular Filtration Rate, PPI=Proton Pump Inhibitors，DM=Diabetes mellitus, CVD=Cardiovascular disease, MetS=Metabolic syndrome, MDS=Magnesium Deficiency Scores.

**Supplement Table 2. Sensitive association between MDS and ED.**

| Analysis | OR(95%CI) | P value |
| --- | --- | --- |
| Unmatched.crude | 3.51 (2.93~4.21) | <0.001 |
| Multivariable.adjusted | 1.61 (1.29~2.01) | <0.001 |
| PropensityScore.adjusted | 1.58 (1.29~1.94) | <0.001 |
| PropensityScore.Matched | 1.47 (1.17~1.85) | 0.001 |
| Weighted.IPTW | 1.67 (1.41~1.97) | <0.001 |
| Weighted.SMRW | 1.84 (1.57~2.15) | <0.001 |
| Weighted.PA | 1.56 (1.23~1.97) | <0.001 |
| Weighted.Ow | 1.56 (1.18~2.08) | 0.002 |

**Supplement Table 3. Demographic and clinical parameters according to OS.**

| Variable | Total | Alive | Dead | P value |
| --- | --- | --- | --- | --- |
| Age(years) | 44.71±0.38 | 40.71±0.36 | 62.11±0.72 | < 0.0001 |
| Age, n (%) |  |  |  | < 0.0001 |
| <40 years | 40.49(0.02) | 47.87(1.43) | 8.39(1.29) |  |
| >=40 years | 59.51(0.03) | 52.13(1.43) | 91.61(1.29) |  |
| Race, n (%) |  |  |  | < 0.0001 |
| Non-Hispanic White | 73.71(0.05) | 72.55(2.01) | 78.73(2.36) |  |
| Non-Hispanic Black | 9.83(0.01) | 9.56(1.15) | 11.02(1.20) |  |
| Mexican Ameirican | 7.84(0.01) | 8.78(1.10) | 3.75(1.09) |  |
| Other Race | 8.62(0.01) | 9.11(1.27) | 6.49(1.55) |  |
| Marital status, n (%) |  |  |  | 0.35 |
| Solitude | 30.76(0.01) | 30.49(1.26) | 32.13(1.87) |  |
| Cohabitation | 69.13(0.04) | 69.51(1.26) | 67.87(1.87) |  |
| PIR, n (%) |  |  |  | < 0.0001 |
| ＜1.3 | 16.04(0.01) | 16.01(1.15) | 20.72(1.53) |  |
| 1.3~3.5 | 33.89(0.02) | 33.99(1.17) | 43.09(1.68) |  |
| ≥3.5 | 45.13(0.02) | 50.00(1.61) | 36.19(2.10) |  |
| BMI, n (%) |  |  |  | 0.23 |
| ＜25kg/m2 | 29.19(0.02) | 29.95(0.93) | 28.08(1.79) |  |
| 25~30kg/m2 | 40.54(0.02) | 41.40(1.14) | 39.83(2.24) |  |
| ≥30kg/m2 | 28.87(0.01) | 28.65(0.98) | 32.09(1.38) |  |
| Education level, n (%) |  |  |  | < 0.0001 |
| Less than or high school | 43.84(0.02) | 41.40(1.26) | 54.63(2.06) |  |
| Above high school | 56.09(0.02) | 58.60(1.26) | 45.37(2.06) |  |
| Smoking status, n (%) |  |  |  | < 0.0001 |
| Never | 42.93(0.02) | 47.21(1.46) | 24.36(1.76) |  |
| Former | 28.75(0.02) | 24.57(1.07) | 46.99(2.01) |  |
| Current | 28.30(0.02) | 28.23(0.96) | 28.66(2.38) |  |
| Vigorous activity, n (%) |  |  |  | < 0.0001 |
| No | 57.63(0.03) | 54.25(1.42) | 72.34(1.88) |  |
| Yes | 39.49(0.02) | 44.37(1.36) | 18.26(1.78) |  |
| Unable to do activity | 2.88(0.00) | 1.38(0.27) | 9.41(1.41) |  |
| Moderate activity, n (%) |  |  |  | < 0.0001 |
| No | 42.36(0.02) | 41.33(1.16) | 46.96(2.03) |  |
| Yes | 55.68(0.03) | 57.81(1.17) | 46.60(1.91) |  |
| Unable to do activity | 1.91(0.00) | 0.87(0.21) | 6.43(1.11) |  |
| DM, n (%) |  |  |  | < 0.0001 |
| No | 89.63(0.04) | 93.67(0.58) | 72.08(1.48) |  |
| Yes | 10.37(0.01) | 6.33(0.58) | 27.92(1.48) |  |
| CVD, n (%) |  |  |  | < 0.0001 |
| No | 90.98(0.04) | 95.67(0.52) | 70.67(1.85) |  |
| Yes | 8.99(0.01) | 4.33(0.52) | 29.33(1.85) |  |
| Hypertension, n (%) |  |  |  | < 0.0001 |
| No | 65.50(0.03) | 71.55(1.09) | 39.58(1.77) |  |
| Yes | 34.36(0.02) | 28.45(1.09) | 60.42(1.77) |  |
| Anemia, n (%) |  |  |  | < 0.0001 |
| No | 94.99(0.04) | 98.85(0.21) | 91.29(0.74) |  |
| Yes | 2.49(0.00) | 1.15(0.21) | 8.71(0.74) |  |
| Cancer, n (%) |  |  |  | < 0.0001 |
| No | 93.55(0.04) | 96.16(0.41) | 82.52(1.38) |  |
| Yes | 6.38(0.01) | 3.84(0.41) | 17.48(1.38) |  |
| MetS, n (%) |  |  |  | < 0.0001 |
| No | 74.72(0.03) | 78.18(0.88) | 59.63(1.59) |  |
| Yes | 25.28(0.02) | 21.82(0.88) | 40.37(1.59) |  |
| ED, n (%) |  |  |  | < 0.0001 |
| No | 81.36(0.03) | 88.41(0.67) | 50.67(2.34) |  |
| Yes | 18.64(0.01) | 11.59(0.67) | 49.33(2.34) |  |
| Magnesium intake(mg) | 327.77±4.08 | 337.74±4.61 | 284.39±6.41 | < 0.0001 |
| Albumin (g/L) | 43.83±0.08 | 44.23±0.08 | 42.08±0.12 | < 0.0001 |
| Total energy (kcal) | 2665.12±24.69 | 2774.25±29.03 | 2190.19±33.79 | < 0.0001 |
| Group |  |  |  | < 0.0001 |
| 0 | 36.86(0.02) | 41.94(1.80) | 14.76(1.55) |  |
| 1 | 44.50(0.02) | 46.47(1.80) | 35.91(2.08) |  |
| 2 | 4.17(0.00) | 3.95(0.42) | 5.12(0.88) |  |
| 3 | 14.47(0.01) | 7.64(0.57) | 44.21(2.29) |  |

**Supplement Table 4. Sensitive association between MDS and mortarity.**

| Analysis | HR(95%CI) | P(Wald's test) |
| --- | --- | --- |
| Unmatched.crude | 3.57 (2.65,4.82) | < 0.001 |
| Multivariable.adjusted | 1.94 (1.41,2.66) | < 0.001 |
| PropensityScore.adjusted | 1.8 (1.31,2.46) | < 0.001 |
| PropensityScore.Matched | 1.91 (1.31,2.77) | < 0.001 |
| Weighted.IPTW | 2.22 (1.68,2.93) | < 0.001 |
| Weighted.SMRW | 2.4 (1.82,3.16) | < 0.001 |
| Weighted.PA | 1.67 (1.14,2.46) | 0.009 |
| Weighted.Ow | 1.71 (1.1,2.66) | 0.017 |
